# Network of Tourism–Industrial Complex in Cities: Typologies and Implications through a Critical Literature Review

**DOI:** 10.3390/ijerph19094934

**Published:** 2022-04-19

**Authors:** Zhenshan Yang, Shuying Zhang, Jiaming Liu, Huijuan Sun

**Affiliations:** 1 Key Laboratory of Regional Sustainable Development Modeling, Institute of Geographic Sciences and Natural Resources Research, Chinese Academy of Sciences, Beijing 100101, China; yangzs@igsnrr.ac.cn (Z.Y.); liujm@igsnrr.ac.cn (J.L.); sunhj.20b@igsnrr.ac.cn (H.S.); 2 College of Resources and Environment, University of Chinese Academy of Sciences, Beijing 100049, China; 3 College of Applied Arts and Sciences, Beijing Union University, Beijing 100191, China; 4 State Key Laboratory of Resources and Environmental Information System, Institute of Geographic Sciences and Natural Resources Research, Chinese Academy of Sciences, Beijing 100101, China

**Keywords:** urban-industrial complex, tourism urbanization, dynamic mechanism, development models, bibliometric method

## Abstract

Although tourism has increasingly become an important activity with wide influences on the economic, social, and spatial development of a city, knowledge and interest mostly remain on its industrial performance and promotion. The synergy between tourism and city development is largely overlooked in many cases, resulting in suboptimal design and planning of city tourism activities and unfledged potentials of city development. The aim of the paper is to propose a view of tourism–industrial complex based on a synergistic perspective in order to clarify the systematic characteristics of urban tourism in an integrated, sustainable manner. Availing of bibliometric methods and drawing on city/urban tourism literature, this paper proposes a concept of tourism–industrial complex to cover current complicated and various tourism activities that are embedded in cities at diverse levels regardless of social, economic, and spatial factors. Then, four types of tourism–industrial complexes are proposed, including demand-driven, resource-dependent, externally forced, and hybrid-driven models. Due to the networked connectivity of urban tourism, urban backgrounds, tourism industry, and external circumstances all contribute to a coupling the tourism city development system. The results provide theoretical constructs and policy recommendations for optimization and sustainable city and tourism development.

## 1. Introduction

Urban tourism emerges gradually as the society enters the era of urban development, and its advent is later than rural tourism, which is initially a holiday vocation of the middle or upper class in the countryside [1]. Although the Industrial Revolution of the 19th century vigorously promoted urban development, it was mainly for production, trade and government, not for tourism and recreation. Daily entertainment was still dominated by indoor activities at locations such as pubs, theaters, and beerhouses in the middle of 19th century, while outdoor activities were few and mainly in free public places, such as parks and squares, making it difficult to promote large-scale urban tourism activities [2]. It was not until the 1970s that urban tourism came to the forefront, which benefited from the substantial improvement of urban infrastructure, especially transportation and information technology. More fundamentally, tourism was regarded as an economic activity by investors to construct profitable projects, such as resort hotels and cultural blocks [3,4]. Hence, tourism has become a new focus of industrial development in urban development, attracting the input of a large number of land, capital, and labor experts [5,6,7]. However, under rapid development, the pursuit of profit maximization and risk uncertainty are still inevitable, thereby causing the failure of tourism investment projects and even obstacles to a sustainable city.

Urban tourism is a form of tourism defined by geographical space, leading some scholars to regard it as tourism activities within the city [8]. However, due to the dynamic expansion of urban space and booming suburban tourism, it is difficult to completely distinguish the tourism products in the city from those in the suburbs [9]. Therefore, a growing number of scholars believe that urban tourism is the general term of all kinds of tourism activities in and around the city, and its distance is limited to being able to return on the same day [10]. The present studies on urban tourism primarily focus on tourism industrial performance and promotion. Scholars generally discuss planning and management [11,12], driving mechanisms [10], tourism products [13,14,15], markets and tourists [16,17], spatial structure [18], socio-economic and cultural impacts [5,6,19,20], etc. However, the changeable relationship between cities and tourism has not been explored extensively [5]. Tourism and urban development complement each other, and clarifying the interaction between tourism and the city is important for optimal design of tourism activities as well as urban development.

With the urban turn of tourism, Mullins introduced “tourism urbanization” to describe the phenomenon in which some towns in Australia benefitted from the development of the leisure and tourism industry [21], which drove population growth and created a labor market. However, this word is not recognized widely by urban researchers because urbanization refers to the migration of a population from agricultural areas to non-agricultural areas as well as the urbanization of industries and spatial landscapes [22]. It is difficult for tourism to contribute to the above changes [23]. Relevant studies have critically captured the integration of tourist destinations and tourist origins, especially the combination of tourism and consumerism, providing new impetus for industrial development in urban territory [21,24]. However, most studies only emphasize urban development or tourism [25]. There is still much work to be done on how to integrate tourism as an industry and spatial activity into the city and how to improve the overall vitality of the city by relying on tourism development [23]. Synergetics, a rather new interdisciplinary field of research, studies how to realize orderly structure spontaneously through the cooperation of the individual parts of a system. The exploration of the synergistic relationship between cities and tourism is an important perspective from which to understand urban tourism [23], as it can clarify the systematic characteristics of urban tourism and provide ideas for the optimization of urban space.

To embrace the above-mentioned problems, this study aims to put forward a new framework about urban tourism. It particularly includes the following objectives: (1) to conclude previous studies on urban tourism and (2) to understand the systematic characteristics of urban tourism from a synergistic perspective. Specifically, this paper starts with a summary of current work on urban tourism based on a systematic literature review and highlights the necessity to apply a cause-oriented synergistic perspective to urban tourism, which addresses the systemic flaws underlying current urban tourism research. Following this, a tourism–industrial complex framework is introduced from a synergistic perspective to further understand urban tourism and analyze its systematic characteristics, especially typologies and dynamic mechanism. This study contributes to theoretical advancement in urban tourism from an integrated system perspective and promotes the construction of urban tourism in a scientific and sustainable manner.

## 2. Materials and Methods

The literature data analyzed in this paper are collected from the Web of Science Core Collection, the largest comprehensive database of academic resources worldwide, from a period up to November 2021. After defining the titles as (“urban*” OR “city”) AND (“tourism”), a total of 577 articles were obtained by manually eliminating book reviews, book chapters, conference abstracts, some duplicate articles, and items completely irrelevant to the research topic.

The bibliometric method describes, evaluates, and predicts external characteristics of literature materials, such as keywords and authors, through mathematical and statistical methods to analyze the research progress of specific disciplines or knowledge fields [26]. Modern information visualization provides technical support for the dynamic analysis of literature data and gives birth to literature analysis methods represented by Mapping Knowledge Domains [27]. CiteSpace software has become one of the most widely used knowledge mapping tools because of its good data mining function and visualization effect. To analyze the literature related to urban tourism, this paper uses CiteSpace Version 5.8 R3 (64-bit) software, created by Chaomei Chen in New York, The United States.

Timezone map of keywords was adopted to describe the evolution and mutual relationship of urban tourism research. In the timezone map, the more frequent the appearance of the keywords, the larger the nodes will be. The lines between nodes represent the co-occurrence relationship, and their thickness indicates the intensity of co-occurrence.

## 3. Analysis and Discussion of Urban Tourism Research

### 3.1. Systematic Literature Review

#### 3.1.1. Research Stages

**Figure 1:**
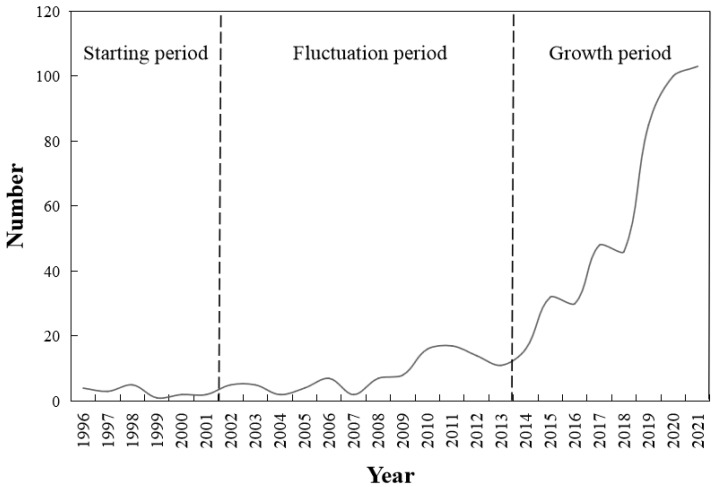
Annual number of publications on urban tourism from 1996 to 2021.

From 1996 to 2021, the number of urban tourism articles published in the journals showed a significant increase (Figure 1). Especially after 2017, researchers paid much more attention to tourism in the context of urban space. Based on the growth rate of articles, following three stages of development were identified:

1. Starting period (1996–2001): Although there are few research achievements during this period, a growing number of studies was conducted in tourism urbanization, which lays a solid foundation for subsequent researches;
2. Fluctuation period (2002–2013): This period witnessed the modest growth in fluctuation of urban tourism research. Along with the development of recreation and leisure industry and a series of international events, such as Poland–Ukraine UEFA EURO, Olympic Games in Britain, and World Expo in Korea, a peak appeared in 2012 [28,29,30]. Regarding urban tourism as a complex system was recognized widely, and relevant studies gradually increased [31,32];
3. Growth period (2014–2021): Research on urban tourism has grown rapidly since 2018, with an annual number of publications even reaching 100. The increasingly close combination of city and tourism provides new impetus for the development of urban industry. In addition to the acceleration of globalization, the COVID-19 pandemic may also be an influencing factor, prompting scholars to explore urban tourist flow in recent years [33].

#### 3.1.2. Main Contents and Theoretical Progress

**Figure 2:**
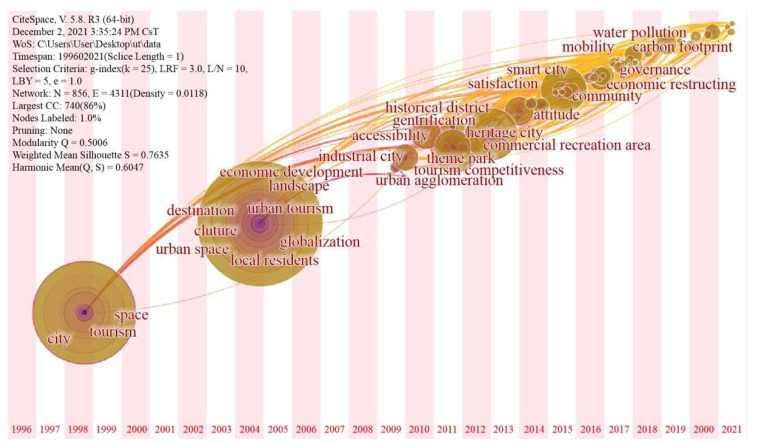
Timezone map of urban tourism research.

The network constituted by numerous nodes (keywords) in timezone map is complex (Figure 2), relating to comprehensive perspectives and contents, such as geography, ecology, management, culture, and marketing. Furthermore, the research themes and theoretical progress also show different characteristics at different time stages (Table 1).

During the starting period (1996–2001), researches on urban tourism followed traditional tourism concepts and ideas. Two of the most important keywords are “tourism” and “city”, with long-term and prominent influences. This period reflects the initial appearance of tourism urbanization. Scholars realized cities can also play the role of tourist destinations and thus carried out tentative discussions.

Keywords showed diversification and systematization during the period of fluctuation (2002–2013). Besides those regarding urban destination, such as “theme park”, “industrial city”, “commercial recreation area”, “urban agglomeration”, and “historical district”, tourism services such as “event tourism”, “accessibility”, and “tourism competitiveness” has also became a significant topic. Scholars began to sort out the logic between cities and tourism during the fluctuation period, and deemed cities as tourist destinations.

During the growth period (2014–2021), studies on urban tourism have been further deepened, showing diversified themes and perspectives. Research priority has shifted from destinations to stakeholders. Keywords such as “mobility”, “governance”, and “community” have emerged. Moreover, social issues also have been emphasized by scholars, especially about environmental protection and sustainable development. “Economic restructuring”, “business improvement”, “water pollution”, and “carbon footprint” have been frequently mentioned. Furthermore, new modes of tourism are hot research issues in this stage. However, most keywords appearing in this period have had limited influencing effects.

#### 3.1.3. Challenging Issues: Relationship between Tourism and City

The theoretical study of urban tourism is experiencing problems in the relationship between tourism and urban development. First, the spatial organization features are not clear. The versatility and diversity make it impossible to regard tourism as a city’s only purpose, and no part of the city should be excluded from tourism [28,37]. Even though functional zones within a city are heterogeneous, and different zones can be easily identified, the multi-zone coupling relationship between urban spatial system and tourism in the aspects of elements, functions, and structures still needs to be further explored [10,55].

Second, the development model needs to be summarized. The development of urban tourism integrates the two-way interaction between cities and tourism [8]. On the one hand, it benefits from the substantial improvement of urban infrastructure [38]. On the other hand, tourism, as an economic activity, has gradually become one of the important sources of urban land development and economic income [6]. In the process of clarifying the interaction between city and tourism, how to choose the appropriate development mode needs to be further clarified to summarize the general rules [11,25,48].

Third, considering the notion that construction of tourist destinations and urban development complement each other and cannot be separated [54], the complexity of a city’s own network puts forward high requirements for sorting out the driving mechanism of urban tourism, which requires comprehensive consideration of internal and external environmental changes [8,25,37].

### 3.2. The Need for a Synergistic Perspective of Urban Tourism

#### 3.2.1. Contradictory or Aligned?

As an important turn of tourism, cities integrate with tourism economic activities to perform specific functions based on a specific spatial system [55]. However, there exist many contradictory but aligned relations in tourism, which generates economic benefits by linking tourism resources and elements scattered in urban space. First, the coexistence of agglomeration and dispersion as well as the contradiction in spatial distribution cause the tourism economy to face unbalanced development although they together constitute the overall form of urban tourism. The city concentrates a wide variety of tourist attractions in a relatively small area and forms different types of tourism precincts according to certain regular spatial organization patterns [56], such as commercial and historical districts. However, not all tourist areas are concentrated in the city center, and the diffusion phenomenon of functional areas dominated by urban agricultural tourism is obvious.

In addition, the openness of cities and the comprehensiveness of tourism determine that urban tourism is not only for tourists. Various themed tours and suburban tours make the identities of citizens overlap with those of tourists and change frequently in daily life and leisure times [57]. For one thing, urban infrastructure, such as transportation, communication, and health care, provides the basis for tourism activities; moreover, tourism service facilities, such as hotels, restaurants, and shopping centers, improve the urban service system. Both of them jointly serve citizens and tourists and promote the interaction of different urban users [10,25].

Admittedly, there are many tourist attractions and economic support in cities [25], and the elements interact frequently, presenting various functions such as economy, society, and culture [8], which lead to the complexity of urban tourism. In turn, in the urban context, the complex relationships of tourism activities also accelerate the connection and flow of elements. In this case, tourism is not only affected by multiple urban factors but also impacts urban development, which is in accordance with synergetics. Tourism and cities can be deemed as individual parts of the urban tourism territorial system, and their synergistic relationship can bring about spatial, temporal, or functional structures. Therefore, urban tourism is both contradictory and aligned, and in the process of exploring it, tourism and city cannot be separated, but the perspective of synergy between the two must instead be sought.

#### 3.2.2. Network of Urban Tourist Destinations

Tourists destinations include tourism supply network and tourism demand network from the marketing perspective. The former means the relationship and interaction among tourism enterprises and organizations; the latter focuses on tourists and activities from the tourism demand side [58]. Previous studies have discussed the network structure [59], influencing factors [60], and evolution [61], which proves that network structure accelerates the development of integrated and competitive tourist destinations [58], and urban tourism destinations are no exception.

**Figure 3:**
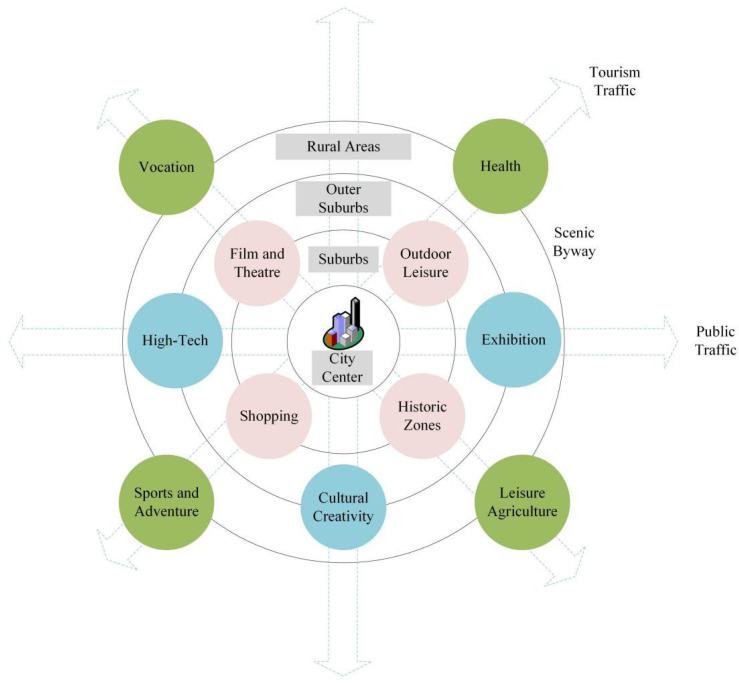
Network of urban tourist destinations.

The network of urban tourist destinations is a dynamic structure formed by the interaction of the elements involved in tourism activities in the man–land relationship system within the urban space [55]. Under the trend of urbanization, tourists take modern urban facilities as the core to participate in tourism activities in urban space, driving the growth of the population and creating the vigorous labor market [6,37]. Therefore, urban tourist destinations are not only limited to the spatial network of tourism activities but also constitute a complex and diversified industrial system (Figure 3). Despite related studies on urban tourism and that urban tourism destinations are abundant, still, there is limited research on the network of urban tourism destinations from the perspective of a synergistic relationship between tourism and city.

## 4. Towards Tourism–Industrial Complex in Cities

### 4.1. Spatial Elements of Urban Tourism

**Figure 4:**
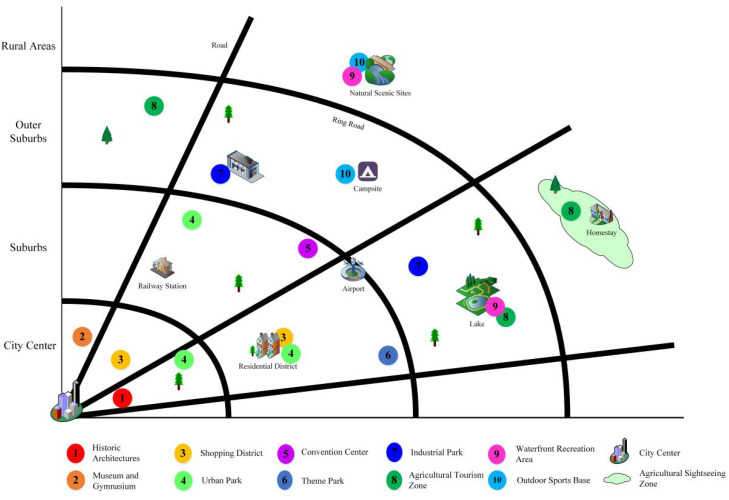
The distribution of tourism space in the city.

Urban tourism is closely related to urban economy and culture. In addition to traditional tourism products, such as sightseeing and shopping, tourism is also developed based on business, exhibition, science and education, leisure, vacation, festivals, and other activities [62,63]. The places that organize these activities are tourist attractions, such as historical and cultural core areas, museums, waterfront areas, theme parks, and exhibition centers [64]. The city concentrates a wide variety of tourist attractions in a relatively small area and forms different types of tourism precincts according to a regular spatial organization pattern [65,66], such as recreation business districts, commercial blocks, and historical core areas. Therefore, the urban tourism territory is a complex system (Figure 4). However, not all tourist precincts are distributed in the city center; the spatial diffusion phenomenon of functional areas dominated by leisure agricultural tourism is obvious [67].

Under the influence of historical culture, historical and cultural buildings, museums, and gymnasiums are mainly located in the central city [68]. Shopping blocks, business districts, and urban parks mainly serve urban residents and are mostly located in downtown areas or near residential areas [69]. Theme parks and exhibition centers are located in the suburbs of the city due to large area and late construction time [70]. With the diversified development of tourism forms, industrial parks located in the outer suburbs are also deemed as tourism resources [71]. The process of urban–rural integration makes leisure agricultural tourism become an important part of urban tourism, attracting a large number of residents and tourists because of farming landscape and country life [72]. The waterfront areas built by rivers and lakes and outdoor sports bases enrich recreation experiences and are located at the edge of the cities [73].

### 4.2. Classification of Urban Tourism–Industrial Complex

This paper divides the development models of the tourism–industrial complex in cities into four typical categories: the demand-driven model, resource-dependent model, externally forced model, and hybrid-driven model. The first three types are driven by the growth of the urban demand market, tourism supply market, and external environment respectively, while the last type is affected by the joint action of multiple factors without obvious leading power.

#### 4.2.1. Demand-Driven Model

Along with increasing public demand for leisure, tourism industry in cities has been praised highly. In some small towns, the target market of tourism may not be local residents but tourists from adjacent cities, which is the objective effect brought by the overall urbanization [9]. Therefore, urban tourism relies on the local and non-local markets. However, the demand of urban tourism in big cities becomes larger with the improvement of living quality [20,47,49]. Building livable cities has been becoming the goal of regional, national, and even international cities. In big cities, residents share urban resources and facilities with foreign tourists, showing the perfection of urban functions and the normalization of recreation [57]. Small cities have small population scale and weak service functions; hence, urban tourism is extroverted and mainly aims at obtaining economic benefits.

#### 4.2.2. Resource-Dependent Model

Tourism resources are the decisive factor for tourists to choose destinations and provide the basis for sightseeing, leisure, and cultural activities. The resource-dependent model means that the supply market represented by highly attractive natural or cultural tourism resources occupies a dominant position in tourism–industrial complex, which is common in cities of different levels [10]. The spatial distribution of tourism resources plays a guiding role in tourism–industrial complex and UTTS, especially the number, grade, and type of tourism resources, greatly affecting the urban functional zoning [18]. In general, for different cities with the same tourism resources, the higher the city level, the more greatly diversified the stimulated tourism products will be [56,69]. On the contrary, the lower the city level, the more difficult it is to control experiential and professional tourism products. In view of the diversity and fragility of tourism resources, the resource-dependent mode has high requirements for ecological environment and sustainable development.

#### 4.2.3. Externally Forced Model

Tourism is recognized as an industry with a long industrial chain, which is expected to drive the development of multiple industries by direct, indirect, and induced benefits, such as catering and retail industries [74]. In order to realize the multiplier effect of tourism and link various industries to achieve maximum benefits, the government will actively promote the investment and operation of tourism projects, improve public infrastructure and service facilities, and optimize relevant industries [50]. For example, the concepts of “tourism +” and “holistic tourism” put forward in China are the top-level design of the integration of tourism industry, and urban tourism is inevitably promoted by them to have greater potential [75]. In addition, many cities will also be affected by economic globalization, which will be driven by the external atmosphere through frequent contacts and exchanges.

#### 4.2.4. Hybrid-Driven Model

This comprehensive type refers to, in the process of urban tourism development, each driving force playing an important role, and it is difficult to distinguish the leading one. In this mode, urban tourism destinations are generally located in economically developed areas with a high level of urbanization and rich natural and cultural resources. At the same time, the government and all sectors of society attach importance to tourism development, and the relationship between stakeholders is coordinated and in a benign state. The integration of tourism and city involves not only economic growth but also the integration of economic functions and industrial structure [76]. Cities with larger scale, higher grade, and more developed economy have higher functional concentration of industry, commerce, production, and service and more obvious centrality in geographical space [31,58]. Driven by different forces, it is easier to build a stable territory system structure and practice sustainable development path.

### 4.3. Dynamic Mechanism of Urban Tourism–Industrial Complex

**Figure 5:**
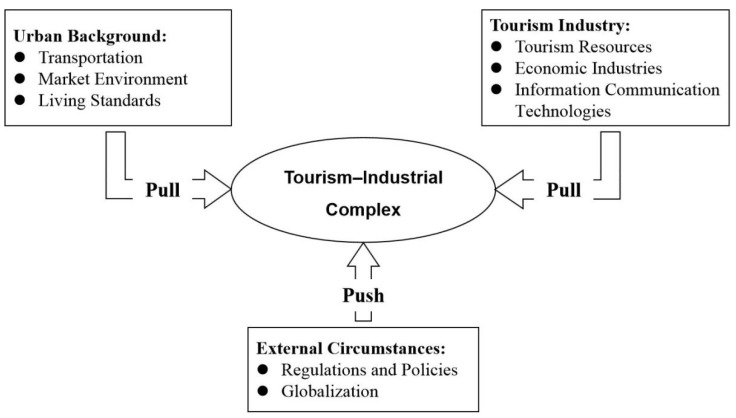
Dynamic mechanism of the urban tourism–industrial complex.

Urban space is the main carrier of tourism, and the elements in the system are constantly connected horizontally and vertically, which promotes the continuous expansion and deepening of the tourism industry [7]. As the intensity of interaction between cities and tourism increases, the functional layout and industrial structure of the tourism–industrial complex are constantly adjusted [77]. At the same time, the external environment represented by regulation and policy and globalization also play significant roles in the evolution of UTTS [55]. Therefore, the tourism–industrial complex is jointly driven by factors such as urban background, tourism industry, and external environment (Figure 5). Among them, urban background and tourism industry are internal pull forces, and external environment is thrust force.

#### 4.3.1. Urban Background

- Transportation is a key element for the formation and development of a tourism–industrial complex [78,79], and the main functions include: (1) connecting cities with tourist origins and destinations; (2) connecting the central city with the outer suburbs; and (3) providing urban traffic services and tourist routes. Tourism transportation networks determine the accessibility and mobility of a tourism–industrial complex, affecting the relationship between the complex and external systems as well the internal elements of the complex [43,80]. Different spatial relations, such as mutual connection, promotion, and restriction, affect the spatial structure formation and evolution of the tourism–industrial complex [81,82].
- Market is an effective method of resource allocation and utilization. The effect of market allocation on urban economy has led to the formation of tourism industry, which can respond to the changes of tourism supply and demand in a timely, accurate, and flexible manner [23,42]. Due to the large number of people and information flows, urban economic and social environment is always in an active state, and the industrial progress represented by commercial development absorbs various production and consumption factors, forming a good opportunity for the development of tourism [10]. The agglomeration and flow of market elements strengthen mutual connections, which causes competition and cooperation and promotes the continuous evolution of the tourism–industrial complex and urban tourism.
- The improvement of living standard and the rise of consumer city rapidly promote urban tourism. The diversification of urban functions forms a multi-functional regional system of industry, production, residence, commerce, and service in leisure times. The increasing income provides a material basis for residents to travel frequently and in short distances. Urban tourism conforms to the market demand of the consumption era [21] and is also a new driving force for the tourism–industrial complex and urban tourism [11,12]. In particular, big cities with higher income level are the main market source of suburban tourism and make an outstanding contribution to urban peripheral tourism.

#### 4.3.2. Tourism Industry

- Tourism resources are the object of tourism activities and the survival basis for tourism. Urban tourism resources both serve the daily recreation of urban residents and non-locals [57]. Most urban areas are dominated by cultural landscape resources involving historical and cultural sites, modern architectures, food and shopping places, festival events, etc. The suburbs include mostly natural landscape resources, with relatively little human intervention and obvious natural landscapes. Although suburban tourism represented by rural homestays has reduced the threshold of urban tourism to a certain extent, with urban rapid development and anti-urbanization, the concept of boutique tourism has attached importance to constantly improve the quality of urban tourism [9].
- Tourism has formed various direct or indirect relations with economic industries. In terms of vertical industry, the urban tourism industry chain relates to nearly all of the economy industries, including basic support industries, such as agriculture, forestry and fishing, manufacturing, and transportation, and directly related industries, such as hotels and travel agencies, as well as entertainment-driven industries, such as film and television industry, and the Internet industry [83]. Hence, the tourism-industrial complex is closely related to the economic structure of the city. In addition to traditional tourism projects, such as sightseeing and shopping, more tourism activities are carried out based on business, exhibition, education, leisure, vacation, and festivals [62,63], thus forming tourism agglomeration area and constituting basic elements of a regional system.
- Information communication technologies cover almost every link of the tourism supply chain in smart tourism, which is crucial to the service connection and improvement of the tourism–industrial complex. Traditional stakeholders, such as hotels, restaurants, and travel agencies, should improve work efficiency in the process of big data collection, storage, analysis, and management. At the same time, cooperation among upstream and downstream industries [84,85,86], application of emerging technologies based on the Internet of Things, virtual reality, augmented reality, and Tourism Satellite Account also contribute to creating urban tourism destinations for participatory sensing systems [87,88,89,90].

#### 4.3.3. External Circumstances

- Regulations and policies support greatly affects the level of urban tourism development. Only by making unified planning, utilizing urban resources, improving service efficiency, ensuring orderly and healthy market environment, and creating a good urban atmosphere can cities promote the sustainable development of tourism.
- Globalization promotes the increasingly close exchanges between cities, thus driving the evolution of the tourism–industrial complex. Since the 1990s, economic globalization and integration have greatly increased the scope and contents of exchanges worldwide. The participators have shifted from countries to cities. Moreover, the increased accessibility of long-distance transportation, including international flights and visa procedures, has greatly encouraged people to travel to foreign cities.

## 5. Discussion

As an important social and economic activity, urban tourism has become an unavoidable problem in the development of many cities. Tourism brings both opportunities and new problems to urban development. Due to the extensive economic driving role of the tourism industry, cities tend to take this opportunity to revitalize the economic network, expand and deepen the industrial chain, and promote the overall development of urban economy [52]. However, in view of the complexity of the urban economic system, the relationship between tourism and urban development is also worth discussing [37]. In modern urban tourism, how to shape the synergistic relationship between the tourism industry and city development is a difficult problem faced by urban tourism. This study puts forwards the tourism–industrial complex from a synergistic perspective of tourism and urban development, which provides theoretical constructs and policy recommendations for optimization and sustainable development in urban tourism destinations.

Even though tourism cannot be regarded as the standard for classifying city types or functional zones [91], the driving effect of tourism on cities has indeed given birth to a series of tourist cities. A city’s versatility and diversity make it impossible to use tourism as its sole purpose, and no part of the city should be excluded from tourism [91]. Besides the possibility of attracting visiting relatives and friends for a large urban population [20], the reasons for development also include public services as a tourist destination. Therefore, the potential source market of urban tourism is huge, which can meet the diversified needs of both general tourists and special groups, such as the elderly, teenagers, and business travelers. It is worth emphasizing that tourist cities are heterogeneous. Even if a city famous for events, cultural exhibitions, heritage sites, customs, and styles can be recognized by the public, it is difficult to define in concept [20]. Furthermore, tourists’ activities in the city often have multiple purposes, and tourists make extensive use of urban facilities. In this way, urban tourism activities have become one of the most important causes of urban population tidal changes and also cause problems for urban infrastructure construction. Based on the above reality, this paper puts forward the concept of the tourism–industrial complex to understand the comprehensive effect of tourism and urban development under the synergistic framework.

Furthermore, several key observations on promoting an urban tourist destination are as follow: First, the tourism–industrial complex accords with the general principles of urban economic development, such as location theory and land tax theory [8,55,76], but also has a certain mutability due to industry innovation. It is necessary to identify different urban functions and services, which is helpful to clarify the multi-layer coupling relationship between an urban territorial system and tourism in aspects of elements, functions, and structures in an urban tourism destination. Second, with the increase of tourism activities and the large influx of visitors, urban tourism may become the most uncertain aspect of the urban environment. Tourism activities are accompanied by a large amount of water and energy consumption, which causes indirect damage to the urban ecological environment under the influence of non-local residents and has time fluctuation [6,30,43]. In order to achieve sustainable development, an urban tourism destination needs to pay attention to the ecological and environmental effects brought by tourists, define the environmental carrying capacity, and implement strict monitoring and management of the ecological environment [39]. Third, the optimization and measurement of the urban tourism industry chain is also the focus of urban development in the future. As we all know, tourism is an activity with a very wide industrial chain, having some specific links with other industries in the city. At present, the city has not thoroughly revealed and measured the tourism industry chain [12]. If this problem is solved, it will be possible to answer the driving role of urban tourism on urban economic development more clearly so as to summarize the best development model of tourism development under different urban backgrounds.

## 6. Conclusions

This paper reviews the studies of urban tourism by adopting a bibliometric analysis. According to the research contents and theoretical evolution of urban tourism, the findings reveal the challenging issues of the relationship between tourism and urban development. Then, the concept of the tourism-industrial complex, and its four typical models and dynamic mechanism were, respectively, explaining to understand urban tourism from a synergistic perspective. This study is beneficial to clarify the synergistic relationship of tourism and cities and offer a feasible way to optimize and promote urban sustainable development.

To study tourism in the city and explore the city from tourism are the general requirements for future urban tourism research and practice, which requires scholars not only to apply urban-related studies to urban tourism research but also to explore urban development scenarios and optimization methods based on tourism activities so as to explore a better combination model of city and tourism in practice. Future research needs to be improved in the following aspects: First, in order to guide the practice scientifically, it is an important direction of research to build the theoretical framework of the tourism–industrial complex based on the reference of an urban spatial system and tourism economic system. Second, from the dimensions of city and tourism scale and grade, the systematic study of the tourism–industrial complex is also a future research trend. Combined with the existing urban tourism activities and spatial-temporal evolution law, it is worth discussing to evaluate the development suitability and construction conditions of the tourism–industrial complex. Third, urban sustainable development needs to pay attention to the ecological and environmental effects of urban tourism.

## Figures and Tables

**Table 1:** Main theoretical progress and typical achievements.

Phases	Research Features	Examples of Achievement
Starting period (1996–2007)	The important position of the city as a tourist destination, addressing tourism, urbanization and systematic structure	The visitor economy and the growth of large cities [20]; The adventure of urban tourism [34]; Engaging postmodern urbanism [35]; Tourism urbanization [36]; Urban tourism as a system [37].
Fluctuation period (2008–2017)	Tourism development of representative cities or specific tourist sites	Hotels in urban tourism destinations [13]; Pro-poor tourism in a first-world urban setting [38]; Urban night tourism [39]; Tourists in big Polish city [18]; Second home and urban landscape pattern [40]; Dual tourist city [41].
	The contribution to spatial regeneration and urban planning	Culture-led regeneration [16]; Urban tourism and its contribution to economic regeneration [42]; Research agenda for Australian urban tourism [43]; Small island urban tourism [14]; Waterfront redevelopment and event tourism [44].
	Tourism service in urban context	The hotel and the city [15]; Tourism and urban public transport [17]; Smart city and smart tourism [11].
Growth period (2018–2021)	Stakeholders involved in urban tourism	Stakeholder perspectives on urban sustainable tourism [45]; Participatory urban tourism planning [12].
	The problem of urban tourism	Sustainable tourism [46]; Overwhelmed city [47]; Urban and rural tourism under COVID-19 [48]; (Re)creating spaces after earthquakes [49]; Urban proximity and wine tourism [50].
	Emerging models and new paths	Creativity in urban tourism [51]; Urban heritage and cultural tourism [52]; Sharing economy [53]; Work, life, and leisure in an urban ecosystem [54].

## Data Availability

The data supporting the reported results in the present study will be available on request from the corresponding author.
